# Tumor microenvironment differences between primary tumor and brain metastases

**DOI:** 10.1186/s12967-019-02189-8

**Published:** 2020-01-03

**Authors:** Bernardo Cacho-Díaz, Donovan R. García-Botello, Talia Wegman-Ostrosky, Gervith Reyes-Soto, Elizabeth Ortiz-Sánchez, Luis Alonso Herrera-Montalvo

**Affiliations:** 1 Neuro-oncology Unit, Instituto Nacional de Cancerología, Av. San Fernando 22. Col. Sección XVI. Tlalpan, 14080 Mexico City, ZC Mexico; 2 Research Unit, Instituto Nacional de Cancerología, Av. San Fernando 22. Col. Sección XVI. Tlalpan, 14080 Mexico City, ZC Mexico

**Keywords:** Tumor microenvironment, Brain metastases, Cancer

## Abstract

The present review aimed to discuss contemporary scientific literature involving differences between the tumor microenvironment (TME) in melanoma, lung cancer, and breast cancer in their primary site and TME in brain metastases (BM). TME plays a fundamental role in the behavior of cancer. In the process of carcinogenesis, cells such as fibroblasts, macrophages, endothelial cells, natural killer cells, and other cells can perpetuate and progress carcinogenesis via the secretion of molecules. Oxygen concentration, growth factors, and receptors in TME initiate angiogenesis and are examples of the importance of microenvironmental conditions in the performance of neoplastic cells. The most frequent malignant brain tumors are metastatic in origin and primarily originate from lung cancer, breast cancer, and melanoma. Metastatic cancer cells have to adhere to and penetrate the blood–brain barrier (BBB). After traversing BBB, these cells have to survive by producing various cytokines, chemokines, and mediators to modify their new TME. The microenvironment of these metastases is currently being studied owing to the discovery of new therapeutic targets. In these three types of tumors, treatment is more effective in the primary tumor than in BM due to several factors, including BBB. Understanding the differences in the characteristics of the microenvironment surrounding the primary tumor and their respective metastasis might help improve strategies to comprehend cancer.

## Background

The tumor microenvironment (TME) consists of cellular and noncellular components. Cancer and noncancerous cells, immune cells, blood and lymphatic vessels, and niche cells belong to the cellular component of TME. The noncellular component is encompassed by cytokines, chemokines, mediators, and growth factors and can influence and be influenced by cancer cell growth [1]. Further, the extracellular matrix (ECM) is an element of TME. Metastatic spread is a process wherein cancer cells move from their primary location to a distant site, colonizing and growing in a new location, and is considered a cancer hallmark [2]. The tumor and the microenvironment (ME) that surrounds it are necessary to initiate a series of steps to invade, colonize, and grow in a distant tissue for induce metastasis [1].

Early research on TME in metastases dates back to 1889 with Stephen Paget’s theory of “seed and soil,” wherein seeds (tumor cells) prefer to grow on a different soil (organ), i.e., ME. This theory has been a cornerstone for the development of anti-angiogenic and immunological therapies directed toward TME [3–5]. The ability of a cell to form a tumor is context dependent, where one environment may promote tumor growth but another will not [6]. Therefore, it is vital to understand the ME more completely.

For metastasis to occur, several steps should transpire: (1) Invasion (outside the basement membrane) by the promotion of cell motility, induction of epithelial-to-mesenchymal type transition (EMT), and secretion of molecules that modulate ME, (2) intravasation into local blood and lymphatic vessels, (3) survival and transit of cancer cells to the circulation/lymphatics, also known as circulating tumor cells (CTCs), (4) arrest/arrival and extravasation at a secondary or foreign tissue, and (5) colonization at secondary sites. These steps in which tumor cells are established at another cell niche are not an intrinsic program. Metastasis is a complex and multifaceted process that has an influence on the tumor cells (mutations, epigenetic changes, and characteristics) as well as on the availability of growth factors, interaction with other tumor cells, and new surrounding ME [1].

Brain metastases (BM) are more frequently observed in everyday practice, occurring in approximately 20% all patients with cancer [7]. The present review discusses contemporary medical literature involving differences between TME in melanoma, lung cancer, and breast cancer in their primary site and TME in their BM. We describe general concepts such as the primary TME, exosomes, EMT and mesenchymal-to-epithelial type transition (MET), CTC, and TME in BM.

## The primary tumor microenvironment

The cellular component of TME includes cancer and noncancerous cells, immune cells, mesenchymal stem cells, endothelial cells, niche cells, cancer-associated fibroblasts, and adipocytes that can promote tumor growth [8]. Mesenchymal stem cells are adult stem cells that can be isolated from the bone marrow; are positive for CD105, CD73, and CD90; and can differentiate into osteoblasts, adipocytes, and chondroblasts [9]. Mesenchymal stem cells support tumor growth through immunosuppression by downregulating the surface HLA class I and antigen-specific T cell recognition of cancer cells by cytotoxic T lymphocytes (CTLs) in vitro [10]. In addition, they suppress the proliferation, activation, and effector functions of CTLs through the generation of adenosine [11].

Immune cells in TME are important. An inflammatory infiltrate is essential for tumor development. The immune system selects cancer cells and helps them escape the immune surveillance system [12]. Cancer-associated macrophages, mast cells, monocytes, natural killer cells, and the innate immune system preserve carcinogenesis because the proinflammatory ME mediated by interleukins (ILs), such as IL-6, and tumor necrosis factor (TNF) activate the nuclear factor (NF)-kB, which regulates other transcriptional factors for EMT to ensue [3].

Secretion of IL-8 by cancer-associated macrophages is one of the most relevant factors in EMT [3]. Cancer cells that undergo EMT and appear mesenchymal (m-cars) promote metastasis and neovascularization within the tumor. Angiogenesis is fundamental because it provides nutritive components and serves as a metastatic pathway [3]. Vascular endothelial growth factor (VEGF)- and VEGF receptor 2 (VEGFR2)-mediated signaling play an important role in angiogenesis [13]. Additionally, oxygen is an essential factor for the occurrence of EMT.

Low oxygen regions within the tumor generate hypoxia-induced factor (HIF) expression. HIF1 and HIF2 promote the expression of other transcription factors including Twist, Snail1, Zeb-1, BMI1, and Notch [3]. Twist1 is associated with the acquisition of stem cell properties and enhancement of metastasis [14, 15]. Hypoxia generates the differentiation of m-cars into endothelial cells. Moreover, m-cars promote the process of angiogenesis by secreting VEGF, IL-8, and fibroblast growth factor (FGF), improving the nutritive and oxygenation conditions of the ME, and promoting the metastatic cascade, because the new vessels are permeable to both cells and macromolecules [16, 17]. VEGF stimulates vascularity and miRNA-105 secretion that interrupts the zonula occludens-type cell junctions in the endothelium as well as the interaction with the α2β1-Cadherin integrin complex, which favors the contact of the metastatic cells with the endothelium [16, 17]. Once m-cars are disseminated into the circulatory system as CTCs, it is crucial that they follow the continuous endothelial signals for their survival. Signals including the epidermal growth factor (EGF), transforming growth factor beta 1 (TGF-β1), and thrombospondin 1 mediate the proliferation and quiescence of cancer cells during dissemination [18].

TME promotes lymphangiogenesis, which in turn promotes cancer cell dissemination. Lymphangiogenesis and the remodeling of lymphatic networks significantly enhances metastasis by secreting VEGF-A/C/D, IL-1β, FGF, and periostin and activating the sympathetic nervous system [1].

### Exosomes

Exosomes are 30–200-nm membrane vesicles filled with proteins, soluble factors, ribonucleic acid (RNA), or micro-RNA (miRNA). Exosomes can be secreted by some cancer cells to communicate with other cells. Exosomes are capable of inducing ME changes in a distant cellular niche, including the cellular matrix, and inducing adhesion molecule and integrin expressions [18]. In cell lines, compared with non-BM cell-derived exosomes, a dysregulation in miRNAs and proteins in BM cell-derived exosomes has been demonstrated; increased adhesion and invasion properties in non-BM cells were observed when they were incubated with BM cell-derived exosomes [19]. Some long noncoding RNA (lncRNAs) are enriched in exosomes, whereas their endogenous expression is low. Interestingly, microRNAs perform cell-independent biogenesis within cancer cell exosomes [17].

### miRNA

miRNA are small noncoding RNA molecules, ranging from 20 to 25 nucleotides in length, that can function as gene regulators by the inhibition of target mRNA translation and deregulation of several bioprocesses, such as cell development, cell differentiation, cell proliferation, and apoptosis [20]. Over 2000 miRNA have been identified in humans and regulate approximately 30% of all human genes. miRNA can both inhibit cancer development and enhance oncogenic mechanisms (i.e., oncomiRNA). Table 1 describes specific miRNAs associated with the suppression or promotion of BM and the primary tumor. Metastatic cells harbor an endogenous deregulated expression of miRNA and other noncoding RNA (ncRNA) to promote their mobility and survival [17].

**Table 1:**
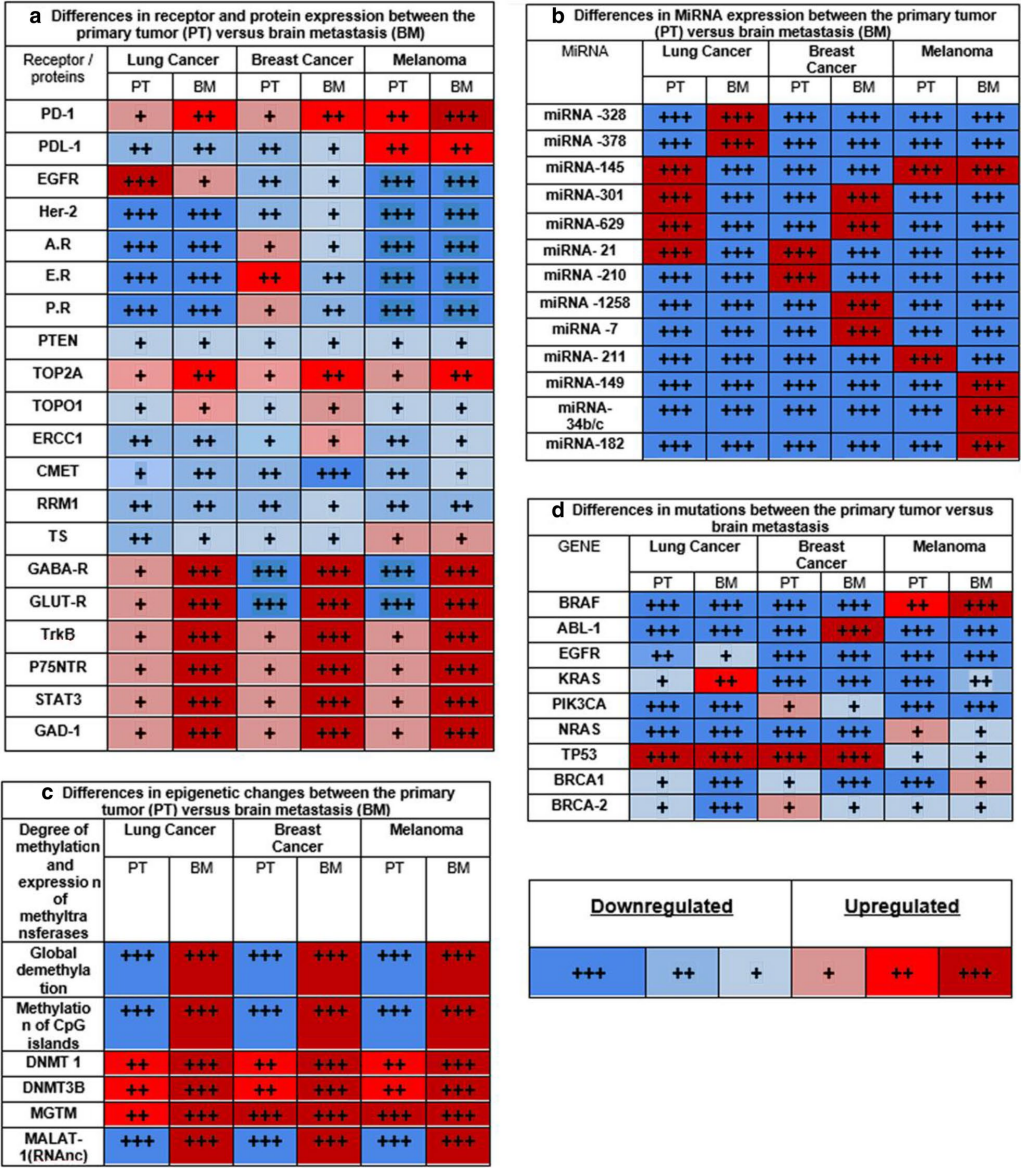
Different protein expression (A), miRNA expression (B), epigenetic changes (C) and mutations (D) between the primary tumor and their brain metastasis

*PD-1* programmed cell death protein 1, *PDL-1* programmed death-ligand 1, *EGFR* epidermal growth factor receptor, *Her-2* human epidermal growth factor receptor 2, *A.R.* androgen receptor, *E.R.* estrogen receptor, *P.R.* progesterone receptor, *PTEN* phosphatidylinositol-3,4,5-trisphosphate 3-phosphatase, *TOP2A* DNA topoisomerase 2-alpha, *TOPO1* topoisomerase I, *ERCC1* repair protein by excision of the cross-complementation group 1, *cMET* tyrosine-protein kinase Met, *RRM1* ribonucleotide reductase catalytic subunit M1, *TS* thymidylate synthetase, *GABA-R* γ-aminobutyric acid, *TrkB* tropomyosin receptor kinase B, *P75NTR* neurotrophin receptor p75, *STAT3* signal transducer and transcription activator 3, *GAD-1* glutamate decarboxylase 1, *MALAT-1* metastasis associated lung adenocarcinoma transcript 1, *DNMT1* DNA (cytosine-5)-methyltransferase 1, *DNMT3B* DNA (cytosine-5)-methyltransferase 3B, *MGMT* O6-methylguanine-DNA methyltransferase, *BRAF* B-Raf protein gene, *ABL-1* serum albumin 1, *EGFR* epidermal growth factor receptor gene, *KRAS* K-ras protein gene, *PIK3CA* phosphatidylinositol 4,5-bisphosphate 3-kinase catalytic, *NRAS* N-ras protein gene, *TP53* p53 protein gene, *BRCA1* breast cancer type-1, *BRCA-2* Breast cancer type-2 [2, 10, 21, 22, 24–26, 28, 32–38, 42, 43, 45, 46, 50]

In breast cancer cells, several miRNA expression profiles have been found to modify the process of initiation, progression, and maintenance [21]. The expressions of miR-31, miR-126, and miR-335 have been shown to suppress metastases in vivo [20]. miRNA-509 [22] is another metastasis-suppressive miRNA that downregulates the expressions of the RhoC and TNF-α genes. RhoC is a well-known oncogene that enhances the migration and invasive ability of cancer cells, and TNF-α increases the blood–brain barrier (BBB) permeability and penetration of cancer cells into the brain [22]. In vivo experiments have shown that low expression of miRNA-509 leads to high secretion of matrix metalloproteinase (MMP)-9 induced by the RhoC and TNF-α pathways, which have not yet been completely elucidated. MMP-9 is a proteinase involved in cancer cell migration and extravasation. Cancer stem cells that are highly metastatic to the brain express lower miRNA-7, modulating the stem-like capacity of cancer cells through *KLF4* expression in breast cancer cell lines and nude mice [23]. KLF4 is one of the induced pluripotent stem cell (iPS) genes required for the maintenance of stemness of progenitor cells [23].

In lung cancer, studies have shown that miR-328 promotes brain metastasis in non-small cell lung cancer, possibly by the upregulation of protein kinase C alpha (PRKCA). High levels of PRKCA have been correlated with an increased migration in cancer cells, which is significantly reduced after the suppression of miR-328 [17] [24]. Moreover, miRNA-378 has been implicated in promoting BM and appears to increase its risk by promoting cancer cell migration, invasion, and angiogenesis [25]. In contrast, miRNA-145 was observed to be low in BM and directly targets mucin 1 (MUC1), a gene associated with the metastatic ability of cancer cells. The suppression of MUC1 decreases the levels of ß-catenin and cadherin 11, which correlate with a decreased cell invasion capacity [26]. miRNA-210 is overexpressed in BM cell-derived exosomes from melanoma, ovarian, and breast cancer cell lines, and a downregulation of miRNA19a and miRNA29c has been observed in BM exosomes [19].

### lncRNA

lncRNA are RNA molecules with a length of > 200 nucleotides. Although they do not encode proteins, lncRNA can alter gene expressions, even after transcription. Furthermore, lncRNA affects mRNA splicing, transportation, and translation. Metastasis-associated lung adenocarcinoma transcript 1 (MALAT1) is overexpressed in some tumors, including non-small cell lung cancer that metastasizes to the brain by inducing EMT [27]. The exact mechanism remains unknown. Moreover, BM from non-small cell lung cancer exhibits a high expression of HOX transcript antisense intergenic RNA (HOTAIR). In vitro studies have reported that HOTAIR enhances cell migration and anchorage-independent cell growth [28]. The exact role and target remain unknown.

Cancer cells secrete noncoding RNA. In addition, tumor-associated macrophages reportedly deliver miRNA-223 to cancer cells through exosomes or microvesicles in vitro and in vivo, which correlates with its invasion ability [29]. miRNA-223 increases the invasiveness in numerous co-cultivated cancer cells, including melanoma, lung, and breast cancer cells [30]. The following aspects have been associated with the secretion of exosomes by cancer cells and tumor-associated macrophages: modulation of the ME to support tumor growth and survival, angiogenesis, evasion of immune surveillance, invasion and metastasis, acquisition of an aggressive phenotype and multidrug resistance through drug efflux from cells [29].

## Epithelial-to-mesenchymal type and mesenchymal-to-epithelial type transitions

A key concept for the occurrence of metastasis is EMT. Most neoplasms originate within an epithelium and then invade the adjacent connective tissue to reach deep tissues (a carcinoma–mesenchymal-type cell). In EMT, malignant epithelial cells express properties similar to fibroblasts and exhibit increased cell adhesion and motility, which facilitates the escape of tumor cells from the primary niche [31]. Metastatic cells from several carcinomas appear less dedifferentiated compared with their corresponding primary tumors [31], which is inconsistent with the EMT-only theory. Therefore, a MET process is required as part of the progression of metastatic tumor formation. EMT is critical for the initial transformation of benign to invasive carcinoma, whereas MET is critical for later stages of metastasis [31]. The greater immunohistochemical expression of E-cadherin and specific connexins of epithelial cells from the primary tumor observed in metastatic cells from patients confirm this notion [32, 33].

The FGF and EGF receptor pathways are essential to induce these cellular changes [31]. Other critical signaling pathways involved in the regulation of EMT include bone morphogenic protein (BMP), TGF, hepatocyte growth factor, Wnt/b-catenin, and Notch pathways [34]. EMT transcription factors (TFs) include Twist1, Snail1, and Prrx1. In both processes, changes in the cellular phenotype occur. In the EMT-like phenotype, EMT TF Prrx1, N-cadherin, and vimentin levels are increased and E-cadherin, occludin, cytokeratin, and claudin levels are decreased. In the MET-like phenotype, E-cadherin, occludin, and cytokeratin levels are increased and EMT TF Prrx1, N-cadherin, and vimentin levels are decreased [35].

## CTC

Approximately 1 million cancer cells per 1 g of tumor enter the circulation daily in patients with cancer [36]; however, only a fraction of these cells survive and reach a distant niche. Therefore, the programming required and intrinsic adaptations that facilitate metastasis represent a significant evolutionary obstacle that tumor cells must overcome [4].

A premetastatic niche is responsible for organizing the ME to welcome circulating cancer cells and serves as a guide to organotropism [1]. These are known as CTCs, which are cells released from primary tumors and metastatic deposits into the bloodstream [37]. The circulome is a functional unit formed by CTCs, immune cells, platelets, any other circulating cells, along with macromolecules and small molecules [38]. Upon entering the bloodstream, the tumor cells are susceptible to severe damage by the shear stress of blood flow and natural killer cells, making it difficult for CTCs to initiate a metastatic cascade.

Initiation of the coagulation cascade and platelet activation mediated by tumor cell tissue can protect CTC by enclosing them in platelet-rich thrombi [39]. Depending on the stimuli and TME, several platelet changes can occur, such as miRNA splicing, protein synthesis, membrane inflammation, and exosome release. In addition, platelets can capture circulating miRNAs from TME or mutant RNAs from tumor cells, suggesting a potential modification in the platelet transcriptome that improves CTC survival [39]. CTCs trapped in such aggregates help their endurance by protecting them from immune surveillance. Two scenarios are proposed for the nature of these platelet aggregations surrounding the CTCs: [1] platelets completely engulf the tumor cells or [2] platelets form homotypic aggregates in the center of the clusters that are surrounded by CTCs in the periphery [39]. In addition, platelets can escort CTCs through the steps of metastatic progression, facilitating adhesion of tumor cells, migration, and extravasation to the secondary site [40].

## BBB

BBB is used to describe the unique properties of the microvasculature of the central nervous system (CNS). CNS vessels are continuous, non-fenestrated vessels containing a series of additional properties that allow them to tightly regulate the movement of molecules, ions, and cells between the blood and CNS [41]. One of the properties of BBB is the tight junctions and presence of efflux transporters to expel harmful molecules. These tight junctions are composed of claudins, occludins and zona occludens proteins, and junctional adhesion molecules [42], with high electrical resistance [43, 44].

Every CTC has to traverse BBB to reach the brain. Cancer cells have to express several proteases to penetrate these junctions [45]. Additionally, these junctions can be destabilized by the expressions of cytokines, chemokines, and inflammatory mediators, including VEGF, basic FGF (bFGF), TGF-ß, IL-1ß, TNF-α, interferon-γ (IFN-γ), CCL2, CXCL8, and prostaglandin-endoperoxide synthase 2 (COX2), by the cancer cells [42, 46–48].

CNS lacks a standard lymphatic drainage system, and the only way for tumor cells to enter the brain is via the bloodstream [40]. The glymphatic pathway is a system specialized in purifying the extracellular materials in the nervous tissue in an influx dependent on the cerebrospinal fluid; it enters the periarterial spaces of the cerebral parenchyma through the channels of aquaporin 4 as a unidirectional way into the perivenous space where the metabolic waste is drained into the systemic circulation [49]. The exact role of the lymphatic system in BM has not yet been elucidated.

To migrate into brain tissue, cancer cells require more time compared with that required to enter other organs. Approximately 48 h is required for lung cancer cells to extravasate into the brain, whereas only 6 h is required for their extravasation into the liver [50]. Breast cancer cells require 2–7 days to extravasate into the brain, whereas melanoma cancer cells require 14 days [51]. Consequently, the arrested tumor cells have to survive within the cerebral vasculature for a significantly longer time compared with that in other metastatic sites [40].

For the occurrence of adhesion to the endothelium by CTC, the function of molecules expressed in BBB and their receptors in CTC, such as selectins, integrins, cadherins, and CD44 and the receptors of the immunoglobulin superfamily (ICAM-1, VCAM1), is of utmost importance [52]. To traverse BBB, angiopoietin-2 has been linked to the early breakdown of BBB and increased colonization of the brain by breast cancer cell metastasis [53]. Cathepsin S mediates in the transmigration by BBB of breast cancer cells through the proteolytic processing of the junction adhesion molecule-B [40]. If BBB were completely disintegrated, it might allow the delivery of effective chemotherapeutic agent doses to BM. However, most chemotherapeutic agents and targeted therapies do not reach BM at sufficient levels [54].

## The tumor microenvironment in brain metastases

The most frequent malignant tumors of CNS are metastases, representing a significant cause of morbidity and mortality in patients with cancer from various neoplasms, mainly from lung cancer, breast cancer, and melanoma [55]. The TME where BM had spread includes cancer and noncancerous cells (i.e., endothelial cells, pericytes, fibroblasts, and immune cells) [56]. The process in which tumor invasion is directed toward the nervous tissue is mediated by different cellular interactions and the brain ME [57]. Reportedly cancer cells activate astrocytes at the metastatic site triggering positive feedback to adapt to the new ME and initiate colonization [17].

The process of cell arrival or nesting in the brain was previously considered a random process. However, the expression of the alpha-2,6, sialyltransferase gene in CTC is associated with a predilection toward BBB, as its protein serves as an adhesion molecule [57]. In breast cancer, the *N*-acetylgalactosaminidase α-2,6-sialyltransferase 5 (ST6GALNAC5) has been identified as a facilitator of tumor cell/brain endothelial adhesion [46]. In melanoma, the membrane-bound melanotransferrin correlates with this ability [58], and in small cell lung cancer, the Rho kinase signaling, involved in the intracellular junction disruption, has been activated in this transendothelial migration [59]. In breast cancer, the chemokine receptor CXCR4 and its ligand CXCL12, also known as the stromal cell-derived factor 1-α, increase vascular permeability and activates the PI-3K/AKT pathway [60]. Factors produced by CTC that predominately aim toward BBB include α-crystallin, ADAM 8 (disintegrin and metalloproteinase domain-containing protein 8), MMP-1, PLEKHA5 (pleckstrin homology domain-containing family A member 5), PT1C (carnitine palmitoyl-CoA transferase), neuroserpin, serpin B2, and CTSS (cathepsin S) [61].

### Transmigration

Transendothelial migration (transmigration) is the process of cancer cells traversing BBB. Factors associated with transmigration include cyclooxygenase COX2 (PTSG2), CXCL12/CXCR4, ST6GALNAC5, CTSS, MMP-1, α-crystallin, angiopoietin-like 4 (ANGPTL4), EGFR ligand heparin-binding EGF (HB-EGF), and VEGF [62]. COX2 activity produces prostaglandin, which increases the BB permeability [63]. Cancer cells produce miRNA-181c, which dysregulates the dynamics of the intracellular actin of BBB via the downregulation of its target gene PDPK1, thus favoring transmigration in vitro and in vivo [61]. Other targets of BBB that serve as tropism factors for CTCs include the expression of E-selectin at brain endothelial cells, which might indicate the site where successful BM occurs [64], and the cell adhesion molecule L1 (L1CAM) [65]. A premetastatic metabolic niche is either required or prepared by CTCs. CTCs secrete miRNA 122 and suppress glucose uptake by neurons and astrocytes. The inhibition of miRNA-122 decreases the incidence of metastases [66].

After traversing BBB, cancer cells secrete various cytokines, chemokines, and mediators, particularly IL-1β, TNF-α, IFN-γ, CCL2, CXCL8, and COX2 [4]. Cancer cells can secrete MMP and other proteases to break down the basement membrane, similar to that occurring in metastases to other sites, and can induce endothelial cell apoptosis [67].

IFN-mediated signaling pathway is essential for antitumor immune response. IFN is produced by numerous immune cells to directly modulate the ME. They also alter gene expression in cancer cells, IFN-stimulated genes (ISGs), and include a family of TFs (IRF3, IRF5, and IRF7), which are the dominant regulators of ISG expression. Metastatic cancer cells downregulate the IFN type I response as a mechanism to promote immune prevention at the metastatic site; therefore, a lack of type I IFN response increases the risk of metastases [33].

In addition to expressing L1CAM and traversing paracellularly into BBB, cancer cells secrete serpins that inhibit the activation of plasmin-mediated astrocytes, thereby preventing the secretion of the pro-apoptotic FAS ligand [68, 69].

Astrocytes maintain homeostasis in the brain ME. Branching astrocytic processes cover most cellular components of CNS, including BBB. Reactive astrocytes release interleukins and upregulate several survival genes in breast cancer cells, such as GSTA5, BCL2L1, and Twist1, promoting resistance to chemotherapy [70]. Metastatic cells then take advantage of the protective function of astrocytes by communication through gap junctions, causing astrocytes to generate survival factors such as IL-1β or CCL2, and by maintaining BBB, which generates another chemoresistance mechanism of BM [71, 72].

The arrest and extravasation of cancer cells result in a strong local activation of astrocytes, detected by the upregulation of glial fibrillary acidic protein (GFAP) as well as by hyperdilation of astrocyte processes [73]. In addition to the elevated expression of GFAP, some astrocytes associated with cancer cells simultaneously regulate the expression of nestin, another marker of reactive astrocytes [74].

A significant consequence of the activation of astrocytes is their ability to secrete factors such as MMP-9. MMP-9 can directly affect the invasion of cancer cells and has proangiogenic and growth-promoting functions in brain tumors through the release of ECM growth factors [73]. A strong expression of MMP-9 and a strong upregulation of the MMP-9 protein in the vicinity of the extravasation of cancer cells, associated with activated astrocytes surrounding the tumor cells, have been observed [73]. Moreover, cancer cells interact with neural stem cells by overexpressing BMP-2, which signals the cells to differentiate into astrocytes [75].

Cancer cells use neurotransmitters as oncometabolites. For example, GABA is used by cancer cells to form NADH [76]. Neurotrophins are endogenous neuron growth factors [such as NT-3, NT-4, nerve growth factor, brain-derived neurotrophic factor (BDNF)]. Cancer cells express the neurotrophin receptor TrkB, which is selectively activated by BDNF, with a possible interaction of HER2+ with TrkB currently being investigated [75]. Microglia form a part of the mononuclear phagocytic system and can respond to the invasive cancer cells via cytotoxic mechanisms. The WNT pathway keeps the microglia active and activates the proliferation of cancer cells. Furthermore, cancer cells express NT-3, which increases the metastatic potential, potentially via microglia-mediated mechanisms [51].

Extravasated cancer cells have to stay in close physical contact to the abluminal surface of the blood vessels to remain viable [77]. VEGF-A influences multiple steps of the metastatic cascade, and anti-VEGF-A therapy can induce long-term dormancy of small, perivascular lung carcinoma metastases [78]. Metastasis is a process rather than a simple endpoint, wherein the arrest of the cancer cells occurs at vascular branch points, cancer cells have to remain close to microvessels, and perivascular growth by early angiogenesis is predominant in lung cancer and by vessel co-option in melanoma [77]. In comparison, breast cancer cells that reach the brain exhibit a GABAergic phenotype similar to neuronal cells, They can use GABA as an energy metabolite and increase cell survival [79, 80].

## Differences between the primary tumor cells and bm

When discussing about metastasis, it is important to consider whether there are any differences between metastatic cells and the primary tumor. Although there are histological and cell markers that persist in metastatic cells, similar to the primary tumor, the metastatic ME is different and probably one of the numerous causes for antineoplastic therapies being less effective on metastatic cells [57, 81]. Of the millions of CTCs, only a few manage to invade another tissue niche [36]; therefore, it can be predicted that these cells must be different. In addition, the variants are represented in the expression of elements that make them more endurable. In breast cancer, the triple-negative subtype shows the highest tendency toward generating BM, possibly due to its resistance to treatment. In clinical practice, Luminal A subtype is most commonly observed with BM [82, 83].

After entering the brain, cancer cells still need to survive the brain ME. Neurons maintain sufficient oxygen by never separating from the nearest capillary for > 40 μm [84]. Cancer cells stay in contact with blood vessels even after extravasation, until VEGF-A induces angiogenesis or the vasculature undergoes remodeling and co-option occurs [77].

### Genomic profiling of BM

Genetic intratumoral heterogeneity within the primary tumor and between primary and secondary (metastatic) tumors has been reported [85]. BM harbors clinically significant mutations that are not detected in the primary tumor in 53% of cases [86]; reportedly, cyclin-dependent kinase (CDK) N2A loss and CDK4/6 amplifications sensitized BM to CDK inhibitors. Another pathway altered in BM includes the PI3K/AKT/mTOR pathway [85]. In addition, regional lymph nodes and other extracranial metastasis samples are not reliable surrogates to detect mutations present in BM [86, 87]. Circulating tumor DNA isolated from the cerebrospinal fluid may serve as a useful biomarker in the future.

### Lung cancer

In non-small cell lung cancer, a higher cumulative incidence of BM has been shown in EGFR-mutant cancer than in EGFR-wild type cancer [88]. EGFR tyrosine kinase inhibitors are currently available as chemotherapeutic agents, which have shown to improve survival in patients with EGFR-mutant cancer. In primary squamous cell carcinoma (SCC), PI3K aberrant tumors showed worse survival and higher incidence of BM. In a recent study [89] that compared BM to its primary tumor in patients with SCC, a whole genome sequencing (WGS) analysis showed heterozygous loss of PTEN (phosphatase and tensin homolog protein gene) in all BM with a gene expression pattern consistent with loss of PTEN.

In patients with lung cancer and BM, a WGS analysis reported that primary tumors showed mutations in genes associated with cell adhesion and motility. BM acquired mutations in the adaptive, cytoprotective genes involved in response to cellular stress including Keap1, Nrf2, and p300 (key players of the Keap1–Nrf2–ARE survival pathway) [90]. Nrf2 is a transcriptional factor that binds to antioxidant response elements (AREs) upon stress and drives the expression of antioxidant genes [90]. Other genetic changes observed in that study included Tp53, Rad54L2, NTRK3, and TARX, which were mutated in the primary tumor; however, these mutations were highly enriched in BM. New targeted therapies for mutations in EGFR, ALK (anaplastic lymphoma kinase), immunotherapy PD-1 (programmed death-1) receptor, and programmed death ligand 1 (PDL-1) have shown promising results in both primary tumor and BM but require further studies.

### Breast cancer

Breast cancer is the second cause of BM. The subtypes associated with a higher risk of BM are triple-negative, basal-like subtype and the HER2 (Human EGF receptor 2)-positive subtype [83]. In patients with breast cancer, whole exome sequencing revealed that BM harbors genomic alterations in the CDK pathway and PIK3/AKT/mTOR pathways, with most of these alterations remaining undetected in the primary tumor. Pairing BM and primary tumors of patients with breast cancer, three genes were found to be frequently methylated and silenced in BM and infrequently methylated in primary tumors: GALNT9 (an initiator of O-glycosylation), CCDC8 (a regulator of microtubule dynamics), and BNC1 (a transcription factor with numerous targets) [91]. Targeted therapies directed toward HER2, mTOR, and EGFR receptors combined with other drugs, such as capecitabine or vinorelbine, are under investigation and might show promising results [87].

### Melanoma

An activating mutation in *BRAF*, an oncogene involved in the MAPK pathway, has been reported in approximately 50% patients with melanoma. A discordance rate in the *BRAF* mutation status of primary melanoma cancer, compared with BM, in patients is reportedly as high as 14% [92]. A paired analysis of primary and BM of patients with melanoma showed that BM had increased the expression of several activation-specific protein markers in the PI3K/AKT pathway [93].

**Fig. 1:**
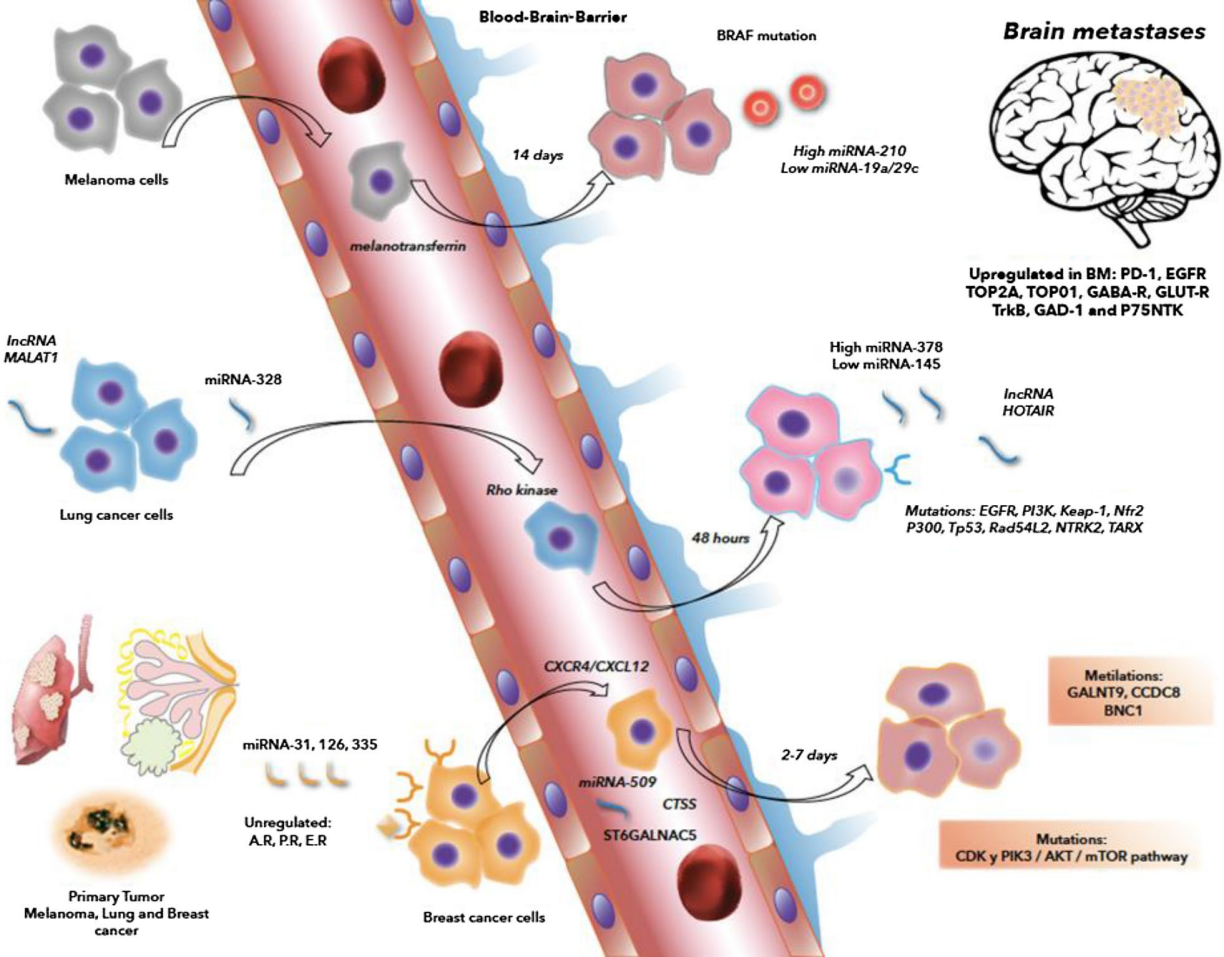
Schematic representation of protein expression, molecular pahtways, facilitators and mutations involved in the genesis of brain metastases from melanoma, breast and lung cancer. Breast cancer cells: Upregulated expression of A.R. (androgen receptor), P.R. (progesterone receptor) and E.R .(estrogen receptor) in the primary tumor. miRNA-509 → Rhoc/TNF pathway → BBB permeability/MMP9 in Circulating tumor cells (CTCs). miRNA-31, -126,-335 suppress metastasic spread. miRNA-7 downregulates KLFM pathway in stem cells. Cathepsin S (CTSS) proteolytic processing of the junction adhesion molecule (JAM). The *N*-acetylgalactosaminidase α2,6 sialyltransferase 5 (ST6GALNAC5) has been identified as a facilitator of tumor cell/brain endothelial adhesion. The chemokine receptor CXCR4 and its ligand CXCL12 increase vascular permeability and activation of the PI-3K/AKT pathway. Lung Cancer cells: Upregulated EGFR, PI3K, Keap-1, Nfr2, P300, Tp53, Rad54L2, NTRK3, and TARX. miRNA-328 upregulates the PKACA pathway. miRNA-378 is upregulated and miRNA-145 downregulated. IncRNA MALAT1 induces EMT and HOTAIR high expression in BM. The Rho kinase signaling, involved in intracellular junction disruption, has been found activated in this transendothelial migration. Melanoma Cancer cells: Upregulated BRAF mutation, induce PI3K/AKT pathway. miRNA-210 was overexpressed in exosomes of BM cells and miRNA 19a and miRNA-29c were downregulated in exosomes BM cells. In CTC, the membrane-bound melanotransferrin correlates with brain endothelial adhesion

Figure 1 shows the differences between the expression of proteins and receptors, mutations, miRNA production, and epigenetic changes among the primary melanoma, breast, and lung cancer cells as well as in those found in BM. Receptors and proteins, such as PD-1, EGFR, TOP2A, TOPO1 GABA-R, GLUT-R, TrkB, GAD-1, and P75NTK, are upregulated in BM. Some receptors are not overexpressed or are unique to a tumor type, such as androgen, estrogen, and progesterone receptors in breast cancer, are downregulated in BM [4, 27, 94–97]. For miRNAs, each of the three cancers overexpresses > 1 miRNA [20, 98, 99]. For lncRNA, MALAT-1 is upregulated in lung cancer and melanoma BM [27, 100]. Epigenetic changes have been reported, demonstrating a global DNA demethylation profile and hypermethylation of the CpG islands in BM from the three cancers in comparison to the primary tumor. The expression of methyltransferases is typically equal or greater in metastases [101–103]. Regarding gene alterations as well as the upregulation of ABL-1 in BM and downregulation of PIK3CA in metastatic breast cancer cells, *BRAF* mutations in melanoma and *EGFR* and *KRAS* overexpressions in lung cancer have been reported [90, 92, 104, 105]. All of these alterations change the expression and function of proteins, receptors, genes, miRNAs, and lncRNAs, and epigenetic mechanisms, thereby upholding a greater aggressiveness of the metastatic disease, leading to low treatment efficacy and poor survival.

Finally, the inflammatory ME is different. In BM compared with the primary tumor site from patients, less infiltration of T lymphocytes (tumor-infiltrating lymphocytes) [106] and lower expression of PDL-1 with higher expression of HLA-1 and PDL-2 have been reported. In addition, tumor-associated macrophages express higher CSF-1, TNF-α, and TGF-β1 levels [71, 97, 106–111].

## Conclusion

TME is fundamental for the progression of cancer to metastatic disease. Tumor cells that establish themselves in the neuronal niche upregulate PD-1, EGFR, TOP2A, TOPO1 GABA-R, GLUT-R, TrkB, and P75NTK as well as miRNA expression, mutations, and specific epigenetic changes. The interaction with the new brain ME renders cancer cells more aggressive and resistant to systemic treatments. BM should be considered as a pathway rather than as a final process to understand its complexity and discover newer ways to approach them.

## Data Availability

Data sharing is not applicable to this article as no datasets were generated during the current study.

## Abbreviations

TME: tumor microenvironment
ECM: extracellular matrix
ME: microenvironment
EMT: epithelial-to-mesenchymal type transition
CTC: circulating tumor cells
BM: brain metastases
MET: mesenchymal-to-epithelial type transition
CTL: cytotoxic T lymphocytes
TNF: tumor necrosis factor
NF: nuclear factor
m-cars: mesenchymal-like carcinoma cells
VEGF: vascular endothelial growth factor
HIF: hypoxia-induced factor
FGF: fibroblast growth factor
EGF: epidermal growth factor
TGF-β1: transforming growth factor beta 1
miRNA: micro-RNA
RNA: ribonucleic acid
lncRNAs: long noncoding RNA
ncRNA: noncoding RNA
MMP: metalloproteinase
PRKCA: protein kinase C alpha
MUC1: mucin 1
MALAT1: metastasis-associated lung adenocarcinoma transcript 1
HOTAIR: HOX transcript antisense intergenic RNA
BMP: bone morphogenic protein
CNS: central nervous system
bFGF: basic FGF
IFN-γ: interferon-γ
COX2: prostaglandin-endoperoxide synthase 2
ST6GALNAC5: *N*-acetylgalactosaminidase α-2,6-sialyltransferase 5
CTSS: cathepsin S
ISGs: IFN-stimulated genes
GFAP: glial fibrillary acidic protein
BDNF: brain-derived neurotrophic factor
SCC: squamous cell carcinoma
WGS: whole genome sequencing
PTEN: phosphatase and tensin homolog protein gene
PD-1: programmed death-1
PDL-1: programmed death ligand 1
HER2: human EGF receptor 2
